# Supplementary material for: Toxicity of Bacillus thuringiensis Strains Derived from the Novel Crystal Protein Cry31Aa with High Nematicidal Activity against Rice Parasitic Nematode Aphelenchoides besseyi
Source: Int J Mol Sci. 2022 Jul 25;23(15):8189. doi: 10.3390/ijms23158189 (PMC9331774; doi:10.3390/ijms23158189)
Supplement: Supplementary file 1 [file ijms-23-08189-s001.zip › ijms-1802221-supplementary.pdf]

International Journal of *Molecular Sciences*

MDPI

Supplementary File

Toxicity of *Bacillus thuringiensis* Strains Derived from the Novel Crystal Protein Cry31Aa with High Nematicidal Activity against Rice Parasitic Nematode *Aphelenchoides besseyi*

Zhao Liang [1, 2†], Qurban Ali [1, 2†], Yujie Wang [2], Guangyuan Mu [3], Xuefei Kan [3], Yajun Ren [2], Hakim Manghwar [4], Qin Gu [1, 2], Huijun Wu [1, 2] and Xuewen Gao [1, 2*]

[1] The Sanya Institute of Nanjing Agricultural University, Sanya 572024, Hainan, China.
[2] Key Laboratory of Integrated Management of Crop Diseases and Pests, Ministry of Education, Department of Plant Pathology, College of Plant Protection, Nanjing Agricultural University, Nanjing 210095, Jiangsu, China; zhaol0619@163.com (Z.L.); 2020202068@stu.njau.edu.cn (Q.A.); yjiewang57@163.com (Y.W.); renyajun0719@163.com (Y.R.); guqin@njau.edu.cn (Q.G.); hjwu@njau.edu.cn (H.W.)
[3] Shenzhen Batian Ecotypic Engineering Co., Ltd., Shenzhen 518057, China; muguangyuan1@sina.com (G.M); xavier2014@163.com (X.K.)
[4] Lushan Botanical Garden, Chinese Academy of Sciences, Jiujiang 332000, China; hakim@lsbg.cn (H.M).
* Correspondence: gaoxw@njau.edu.cn; Tel.: +86-025-8439-5268
† These authors are contributed equally to this study.

Table S1. Bacterial strains used in this study.

Strains	Characteristics	Source or reference
Top10	*Escherichia coli*	TaKaRa Bio Inc.
BL21	*Escherichia coli*	TaKaRa Bio Inc.
GBAC46	*Bacillus thuringiensis*	This study
NMTD81	*Bacillus thuringiensis*	This study
FZB42	*Bacillus amyloliquefaciens*	Stored in the lab

Table S2. Defense related genes primers used in this study.

No.	Gene	Code	Primer(5’–3’)
1	*PR1a*	*PR1a*-F	TCTTCATCACCTGCAACTACTC
		PR1a-R	ATTCATCGGATTTATTCTCACC
2	*PR4*	*PR4*-F	TCTAACGTGCGAGCCACATA
		PR4-R	CTGGATACACTTGCCACACG
3	*LOX1*	*LOX1*-F	GCATCCCCAACAGCACATC
		LOX1-R	AATAAAGATTTGGGAGTGACATATTGG
4	*PBZ1*	*PBZ1*-F	CTACTATGGCATGCTCAAGAT
		PBZ1-R	ATAGAAAGGCACATAAACACAA
5	*PAL1*	*PAL1*-F	ACTACCCGCTGATGACCAAG
		PAL1-R	GCTCTCCTGATCCTGTTGG

Table S3. Proteins primers used in this study.

No.	Protein	Code	Primer(5’–3’)
1	Cry31Aa	*cry31Aa*-F	TGCAGGATCCATGACTAAAAACCACAAGAAAATA
		cry31Aa-R	CGAAGCTTTCACTTATCGTCGTCATCCTTGTAATCTTTTTCGCACCGATAACTT
2	Cry40ORF	*cry40ORF*-F	GCGGGATCCATGTTCACTAATAATGCGAAAA
		cry40ORF-R	GCGAAGCTTTCACTTATCGTCGTCATCCTTGTAATCACTAAGCTGAGTAGTTACTC
3	Cry73Aa	*cry73Aa*-F	TGCAGGATCCATGACCATAACAGTTTTAGATA
		cry73Aa-R	CGGGTACCTCACTTATCGTCGTCATCCTTGTAATCATTTATAAATAAACTATTCA
